# Hepatitis E Virus Infection in Sheltered Homeless Persons, France

**DOI:** 10.3201/eid1806.110632

**Published:** 2012-06

**Authors:** Sylvie Larrat, Stéphanie Gaillard, Monique Baccard, Lionel Piroth, Patrice Cacoub, Stanislas Pol, Christian Perronne, Fabrice Carrat, Patrice Morand, for the French National Agency for Research on AIDS and viral hepatitis HC02 Ribavic Study Team

**Affiliations:** University Hospital of Michallon, Isère, Grenoble, France

**Keywords:** Hepatitis E virus, homeless persons, HEV transmission, autochthonous hepatitis, injection drug use, France, viruses, letter

**To the Editor:** Kaba et al. (*1*) reported a seroprevalence of 11.6% for hepatitis E virus (HEV) among homeless persons in the city of Marseille, located in southern France, and a multivariate analysis suggested that injection drug use (IDU) was an independent risk factor for HEV transmission. We disagree with this reported finding.

We conducted a retrospective subanalysis of results from a multicenter therapeutic trial assessing HEV seroprevalence among HIV/hepatitis C co-infected patients in France (*2*). Serum samples from 84 IDU patients, enrolled during 2000–2002 were stored at −80°C. The mean ± SD age of the patients was 39 ± 4 years; 53 (63%) were men, 19 (23%) were born outside France, and 38 (45%) were living in southern France. HEV antibodies were tested with the same assay as that used by Kaba et al. (*1*), and HEV RNA was detected by using a real-time reverse transcription PCR amplifying open reading frame 3 (*3*). None of the patients had detectable IgM against HEV or HEV RNA. Test results for 3 (3.6%) patients were positive for HEV IgG. Two of them lived in southern France, resulting in a 5.3% (2/38) HEV prevalence for IDU patients living in this region, where HEV IgG prevalence for healthy blood donors has reportedly ranged from 9% to 16.6% (*4*).

The difference between our study, which demonstrated low HEV IgG prevalence in IDU patients, even in southern France, and the results from Kaba et al. (*1*) must be interpreted with caution because there were several epidemiologic differences between the 2 populations. Moreover, there is a risk for false-negative serologic results for HIV patients because of impaired immunity, and the predictive value of serologic testing is probably low because of the artificially low HEV prevalence reported for this population. Despite these limitations, our study suggests that the high prevalence of HEV infection among homeless persons in southern France was not influenced by IDU, but reflected the general epidemiology of HEV in this region.
