# Supplementary material for: Hepatitis E Virus Infection in Sheltered Homeless Persons, France
Source: Emerg Infect Dis. 2012 Jun;18(6):1031–2. doi: 10.3201/eid1806.110632 (PMC3358140; doi:10.3201/eid1806.110632)
Supplement: Related texts

# Reply: Hepatitis E virus infection in sheltered homeless persons, France [letter].

**DOI:** 10.3201/eid1806.111412

**Authors:** Mamadou Kaba, Philippe Colson

**Affiliations:** Timone University Hospital, Marseille, France; and University of the Mediterranean (Aix-Marseille-II), Marseille

**In Response:** The letter of Larrat et al. (*1*) raises interesting questions, but we disagree with the authors’ conclusion. Their study and ours investigated the seroprevalence of infection with hepatitis E virus (HEV) (*1**,**2*); however, the 2 studies examined different populations. We studied homeless persons, for whom analysis of risk factors associated with HEV infection emphasized injection drug use (*2*). This represented independent data: injection drug use was a behavior associated with increased anti-HEV prevalence; a causal relationship between injection drug use and hepatitis E was not inferred. In contrast to our study population, the population studied by Larrat et al. comprised patients who were co-infected with HIV and hepatitis C virus and who reported injection drug use as the route of HIV or hepatitis C virus transmission: a distinctly different population from homeless persons (*1*). It is likely that behavior of HIV-positive and HIV-negative intravenous drug users is not the same. Moreover, late seroconversion, persistent seronegativity, and seroreversion of IgG against HEV have been reported for severely immunocompromized patients, including some infected with HIV (*3**–**5*), which brings up the question as to whether prevalence of IgG against HEV is underestimated among severely immunocompromised persons infected with HIV.

Seroprevalence studies of different populations, especially those with differing immune responses, cannot lead except by chance to the same result. Of note, we recently reported that HEV seroprevalence was 2.3% among injection drug users infected with HIV (*5*).

## References

[1] Larrat S, Gaillard S, Baccard M, Piroth L, Cacoub P, Pol S, Hepatitis E virus infection in sheltered homeless persons, France [letter]. Emerg Infect Dis. 2012;18:1031.2260777510.3201/eid1806.110632PMC3358140

[2] Kaba M, Brouqui P, Richet H, Badiaga S, Gallian P, Raoult D, Hepatitis E virus infection in sheltered homeless persons, France. Emerg Infect Dis. 2010;16:1761–3.2102953810.3201/eid1611.091890PMC3294504

[3] Dalton HR, Bendall RP, Keane FE, Tedder RS, Ijaz S. Persistent carriage of hepatitis E virus in patients with HIV infection. N Engl J Med. 2009;361:1025–7. 10.1056/NEJMc090377819726781

[4] Kenfak-Foguena A, Schöni-Affolter F, Bürgisser P, Witteck A, Darling KEA, Kovari H, Swiss HIV Cohort Study. Hepatitis E virus seroprevalence and chronic infections in patients with HIV, Switzerland. Emerg Infect Dis. 2011;17:1074–8. 10.3201/eid1706.10106721749774PMC3358194

[5] Kaba M, Richet H, Ravaux I, Moreau J, Poizot-Martin I, Motte A, Hepatitis E virus infection in patients infected with the human immunodeficiency virus. J Med Virol. 2011;83:1704–16. 10.1002/jmv.2217721837786
